# Topology Optimization for Hybrid Lattice Compliant Mechanisms with Multiple Microstructures

**DOI:** 10.3390/ma15207321

**Published:** 2022-10-19

**Authors:** Nan Wei, Hongling Ye, Weiwei Wang, Jicheng Li, Fuwei Tian, Yunkang Sui

**Affiliations:** 1 Faculty of Materials and Manufacturing, Beijing University of Technology, Beijing 100124, China; 2 College of Mechanical and Architectural Engineering, Taishan University, Taian 271000, China; 3 College of Engineering, Peking University, Beijing 100871, China

**Keywords:** hybrid lattice, compliant mechanisms, topology optimization, multiple microstructures, ICM method

## Abstract

Hybrid lattice compliant mechanisms (HLCMs) composed of multiple microstructures have attracted widespread interest due to their superior compliant performance compared to the traditional solid compliant mechanisms. A novel optimization scheme for HLCMs is presented using the independent continuous mapping (ICM) method. Firstly, the effective properties of multiple orthogonal and anisotropic lattice microstructures are obtained by taking advantage of homogenization theory, which are used to bridge the relationship between the macrostructure layout and microstructure recognition. Then, a new parallel topology optimization model for optimizing HLCMs is built via a generalized multi-material, recognizing interpolation scheme with filter functions. In addition, the characterization relationship between independent continuous variables and performance of different elements is established. Sensitivity analysis and linear programming are utilized to solve the optimal model. Lastly, numerical examples with a displacement inverter mechanism and compliant gripper mechanism demonstrate the effectiveness of the proposed method for designing HLCMs with various lattice microstructures. Anisotropic lattice microstructures (ALMs) significantly facilitate the efficient use of constitutive properties of materials. Hence, HLCMs consisting of various ALMs achieve superior compliant performance than counterparts comprising different orthogonal lattice microstructures (OLMs). The presented method offers a reference to optimize HLCMs, as well as promotes the theoretical development and application of the ICM method.

## 1. Introduction

Compliant mechanisms (CMs) have attracted widespread attention and become an innovative research hotspot in recent decades because such mechanisms can achieve partial transformation of the displacement and force to greatly reduce the friction, lubrication, and assembly problems due to their deformation [1,2]. CMs are special mechanisms that obtain motions through the deflection of their compliant members. Consequently, the CMs have promising application values in wide fields covering macroscale/microscale manipulation [3], precision manufacturing [4], microelectromechanical systems [5], and vibration control [6]. Abundant design research of CMs has been carried out, and several familiar methods have been proposed, incorporating the kinematics-based method [7,8], building blocks method [9,10], and topology optimization method [11,12,13]. HLCMs with multiple microstructures can make better use of constituent materials to improve the compliant performance with respect to traditional solid CMs. However, HLCMs coupling macroscopic and microscopic optimization are very difficult to achieve due to the huge design space. With the blossoming development of topology optimization, the design of HLCMs has become feasible.

Topology optimization is a progressive concept design method that can fulfill the optimal distribution of materials to minimize or maximize a given objective function under the prescribed constraints in an assumed design domain [14]. Plentiful topology optimization methods have been put forward, mainly including the solid isotropic material with penalization (SIMP) method [15,16], the homogenization method [17,18], the level set method (LSM) [19,20], the moving morphable components (MMC) method [21,22], the evolutionary structural optimization (ESO) method [23,24], the phase field method [25,26], and the ICM method [27,28]. The abovementioned methods have been expanded to design CMs. The seminal work for CMs design can be traced back to [29,30].

Recently, the topology optimization method for CMs research has evolved from single-material to multi-material design and from mono-scale to multiscale design. The early two-material CMs [31] were designed using an ingenious weighted material interpolation, which has been extensively utilized in generating multi-material CMs [32]. CMs with two different materials greatly improved the objective performance compared to the CMs with a single material due to the stiff phase and soft phase being distributed in the load transfer regions, as well as the likewise hinge regions [33]. With the aim of tackling the problem of design variables being too large in multi-material topology optimization, an ordered SIMP interpolation scheme was presented, characterizing the relationship between the elastic modulus and normalized design variables by introducing scaling and translational coefficients [34]. To overcome the point flexure problem with undesired de facto hinges, the parametric LSM was introduced to simultaneously optimize the objective displacement and structural compliance of the multi-material CMs [35]. However, the abovementioned multi-material CMs consist of distinctly different materials, and an obvious interface strength problem exists between any two types of materials, which restricts the improvement of compliant performance.

To surmount this shortcoming, uniform and nonuniform lattice CMs have been proposed to coordinate microstructure selection and macrostructure layout through multiscale optimization. With the premise of evenly distributing the identical microstructures in the macrostructure, the uniform lattice structures are acquired [36,37]. The merits of a uniform design scheme mainly include computational efficiency improvement and excellent connectivity. However, the structure performances are constrained in view of the constitutive materials not being utilized efficiently. Nonuniform lattice structures facilitate a better adjustment of the macro- and microstructures. Therefore, nonuniform lattice CMs have attracted more and more interest. Rodrigues et al. [38] first established a hierarchical optimization model to realize the macroscopic structure layout and microscopic microstructure design, in which the hierarchical calculation did not take full advantage of the design degree and only implemented a compromised concurrent optimization. The compromised optimization issue was conquered by building and solving an approximately constitutive model with tensor decomposition [39]. Multiscale topology optimization has been utilized to design nonuniform CMs with any number of particular microstructures, and the performance of the optimized porous CMs exhibited apparent improvement compared to their counterparts designed using single-scale topology optimization [40]. An adaptive polygonal finite element method is utilized to fulfill the topology optimization of submerged breakwater under fluid–structure interaction, which contributes to the coastal protection [41]. Based on the adaptive geometric components, robust concurrent topology optimization of porous infills and incompressible multi-materials with uncertain load are proved effectively [42,43] and expanded to optimize the coated structure with buckling constraints [44]. The CMs with lattice microstructures were used in the balancer and blade to fulfill tailoring stiffness, which demonstrates that the obtained CMs possess better mechanical performance than the traditional mechanisms [45,46,47]. In addition, studies on the functionally graded materials’ CMs have been applied to enhance the mechanical or geometric advantages with respect to traditional homogeneous CMs [48,49,50].

The above studies mainly focused on the multi-material CMs, gradient lattice CMs, or HLCMs consisting of various kinds of OLMs. Nevertheless, there are few research studies on HLCMs comprising various kinds of ALMs [51,52]. Hence, this paper puts forward a novel optimization scheme for the HLCMs with different microstructures. An HLCM with various ALMs obtains a superior compliant performance than an HLCM with various OLMs because the ALMs could make full use of constitutive materials.

The remainder of the work is organized as follows. In Section 2, effective elastic properties of all kinds of OLMs and ALMs are calculated on the basis of the homogenization theory. Section 3 establishes a parallel topology optimization model through a generalized multi-material recognizing interpolation scheme and gives an elaborate solution procedure. In Section 4, several numerical examples are demonstrated to prove the validation of the current method. Lastly, Section 5 draws the conclusions.

## 2. Effective Elastic Properties of Multiple Lattice Microstructures

Lattice microstructures are increasingly used in wide industrial domains taking full advantage of constituent materials, providing the chance to design novel geometries to satisfy the specified demands corresponding to macroscale physical performances [53]. For the sake of putting into use lattice microstructures for CM design, homogenization theory is introduced to compute the effective elastic properties of lattice microstructures [54,55]. The perturbation theory is utilized to asymptotically expand the elastic governing equation; then, the relationship between the effective elastic tensor and local strain is achieved as follows:

(1) $$D_{ijkl}^{H}=\frac{1}{\left|Y\right|}\int_{Y}\left(D_{ijkl}-D_{ijpq}\frac{\partial \chi_{p}^{kl}}{\partial y_{q}}\right)dY$$

where $D_{ijkl}^{H}$ denotes the effective elastic tensor; $\left|Y\right|$ and $\chi_{p}^{kl}$ represent the volume of the periodic microstructure and local displacement field, respectively; $D_{ijkl}$ denotes the elastic tensor of constitutive material; and $y_{q}$ represents the local microscopic variable. The quadrilateral finite element, which has eight degrees of freedom, is utilized to calculate the effective elastic properties of lattice microstructures.

**Figure 1:**
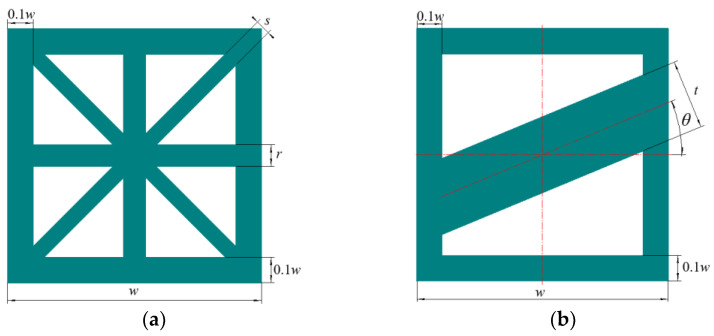
Lattice microstructures with geometric parameters: (**a**) OLM; (**b**) ALM.

For a more convenient discussion, this paper uses dimensionless units. The Young’s modulus of the constitutive material is hypothesized as 1000, and Poisson’s ratio is assumed equal to 0.3. The volume fractions of multiple lattice microstructures are maintained the same at 0.6, so as to coordinate the macrostructure layout and the display effect of microstructures. The effective elastic tensors of various OLMs and ALMs are computed via the above-described homogenization theory. The subsequent research mainly considered regular OLMs and ALMs to design the HLCMs for comparison and verify their feasibility. Figure 1 exhibits the quadrate OLM containing four branches and the quadrate ALM containing a branch, where *w* represents the dimension of the microstructure, the wall thickness is 0.1 *w,* and it is kept consistent. The *r* indicating horizontal branch size and the diagonal branch size *s* decide the configurations of OLMs in Figure 1a. The *t* indicating slant branch size and the slant branch degree *θ* decide the configurations of ALMs in Figure 1b. The effective elastic tensors ***D***^H^ of the prescribed OLM1, OLM2, OLM3, and OLM4 are listed in Table 1. The effective elastic tensors ***D***^H^ of the prescribed ALM1, ALM2, ALM3, and ALM4 are listed in Table 2. In fact, the microstructures can be extended to be more diverse and complex in future work. The effective stiffness matrix ***k***^0^ of the lattice microstructure can be obtained as follows:

(2) $$k_{}^{0}=\int_{Y}B^{T}D^{H}BdY$$

where **B** indicates the strain–displacement matrix.

## 3. Parallel Topology Optimization Formulations for HLCMs

### 3.1. Parallel Topology Optimization Model

**Figure 2:**
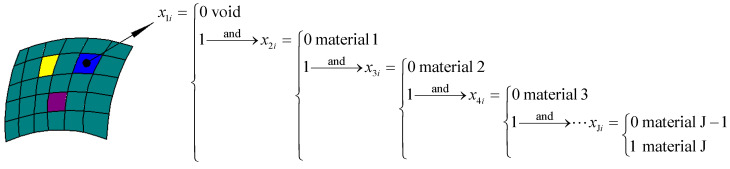
Characterization of multi-material-recognizing interpolation scheme.

A parallel topology optimization model for HLCMs’ design based on the ICM method is formulated by a multi-material-recognizing interpolation scheme. In the multi-material topology optimization process, multiple vital filter functions are incorporated to establish the relationships between different element properties and various kinds of independent continuous topological variables. The objective output displacement of CMs is maximized under a volume fraction constraint. Figure 2 exhibits that an original design domain is divided into a number of finite elements, which characterizes the multi-material-recognizing interpolation scheme. To determine which kind of microstructure is suitable for every element, multiple kinds of topological variables relating to every element are introduced to seek out the optimized macroscopic topology and rational layout of various lattice microstructures. The volume $v_{i}$ and stiffness matrix $k_{i}$ of the *i*-th element in a three-phase domain (two different constituent materials and one void phase) can be identified using various filter functions [56] as follows:

(3) $$v_{i}(x_{1i},x_{2i})=f_{v}(x_{1i})\{[1-f_{v}(x_{2i})]v_{1}^{0}+f_{v}(x_{2i})v_{2}^{0}\}=f_{v}(x_{1i})f_{v}(x_{2i})(v_{2}^{0}-v_{1}^{0})+f_{v}(x_{1i})v_{1}^{0}$$

(4) $$k_{i}(x_{1i},x_{2i})=f_{k}(x_{1i})\{[1-f_{k}(x_{2i})]k_{1}^{0}+f_{k}(x_{2i})k_{2}^{0}\}=f_{k}(x_{1i})f_{k}(x_{2i})(k_{2}^{0}-k_{1}^{0})+f_{k}(x_{1i})k_{1}^{0}$$

where $x_{1i}$ and $x_{2i}$ are the first and second kinds of topological variables with regard to the *i*-th element, respectively; $v_{1}^{0}$ and $v_{2}^{0}$ respectively represent the intrinsic volumes of the first and second kinds of microstructure elements; $k_{1}^{0}$ and $k_{2}^{0}$ indicate the intrinsic stiffness matrices of the first and second kinds of microstructure elements, respectively; $f_{v}(x_{1i})$ and $f_{v}(x_{2i})$ are the first and second volume filter functions, respectively; and $f_{k}(x_{1i})$ and $f_{k}(x_{2i})$ are the first and second stiffness matrix filter functions, respectively. Based on Equations (3) and (4), it can be apparently seen that the first kind $x_{1i}$ confirms the macroscale structure (with or without material), while the second kind $x_{2i}$ recognizes the designated material for every retained element.

A generalized multi-material-recognizing interpolation scheme that can handle any kind of material is proposed by expanding Equations (3) and (4).

(5) $$v_{i}(x_{1i},x_{2i},\cdots x_{Ji})=\sum_{j=2}^{J}\left[(v_{j}^{0}-v_{j-1}^{0})\prod_{q=1}^{j}f_{v}(x_{qi})\right]+f_{v}(x_{1i})v_{1}^{0}$$

(6) $$k_{i}(x_{1i},x_{2i},\cdots x_{Ji})=\sum_{j=2}^{J}\left[(k_{j}^{0}-k_{j-1}^{0})\prod_{q=1}^{j}f_{k}(x_{qi})\right]+f_{k}(x_{1i})k_{1}^{0}$$

where $x_{qi}$ denotes the *q*-th kind of topological variable; J represents the total kinds of prescribed materials; $v_{j-1}^{0}$ and $v_{j}^{0}$ respectively represent the intrinsic volumes of the (*j*-1)-th and *j*-th material elements; $k_{j-1}^{0}$ and $k_{j}^{0}$ represent the intrinsic stiffness matrices of the (*j*-1)-th and *j*-th material elements, respectively; $f_{v}(x_{qi})$ and $f_{k}(x_{qi})$ are the *q*-th kind of volume filter function and stiffness matrix filter function of the element, respectively; and J represents the total material kinds.

Equations (5) and (6) demonstrate that each element corresponds to J kinds of topological variables, but in finite element analysis (FEA), the total number of discretized elements can retain constant. The present scheme facilitates their integration into the generalized multi-material topology optimization using the ICM method [57].

According to the multi-material-recognizing interpolation scheme, the parallel topology optimization model with objective output displacement maximization subject to total volume constraints is mathematically expressed as follows:

(7) $$\begin{array}{l} \mathrm{find}\;\;\;\;x={\left(x_{11},x_{12},\cdots ,x_{ei},\cdots ,x_{\mathrm{JN}}\right)}^{T}\\ \mathrm{maximize}\;u_{\mathrm{out}}\\ \mathrm{subject}\;\mathrm{to}\;Ku=F\;\\ \;\;\;\;\;V=\sum_{i=1}^{N}v_{i}\leq \bar{V}=R_{v}V_{0}\\ \;\;\;\;\;0<\underline{x_{1i}}\leq x_{1i}\leq 1,\;i=1,2,\ldots ,N\\ \;\;\;\;\;0\leq x_{ei}\leq 1,\;e=2,3,\ldots ,J,\;i=1,2,\ldots ,N \end{array}$$

where *u*_out_ denotes objective output displacement; ***x*** represents the total unknown vector of the whole topological variables *x_ei_*; ***K***, ***F***, and ***u*** denote the global stiffness matrix, external force vector, and displacement vector of the study object, respectively; *V* and $\bar{V}$ respectively represent the actual volume and its allowable volume; V_0_ and *R*_v_ are the design domain volume and the prescribed volume fraction, respectively; and N denotes the total number of finite elements. Besides, $\underline{x_{1i}}$ is the lower limit of the first type of topological variables and is set as 0.0001 to circumvent numerical singularity.

### 3.2. Sensitivity Analysis and Solution

An approximately explicit expression of the objective output displacement is the greatest challenge. For computational convenience, the power functions are utilized for all various kinds of filter functions as follows:

(8) $$f_{v}(x_{ei})=x_{ei}^{}\;e=1,2,\ldots ,J$$

(9) $$f_{k}(x_{ei})=x_{ei}^{p}\;e=1,2,\ldots ,J$$

where *p* represents the filter power factor of the element stiffness matrix and is allocated as 3 to fulfill faster convergence and better material distribution.

For the purpose of acquiring the approximately explicit expression of the objective output displacement, the adjoint method [58] is applied to solve partial derivatives of the objective output displacement with respect to various kinds of topological variables [59]. Since the external load vector ***F*** is not dependent on topological variables, the partial derivative of the equilibrium equation in Equation (7) in regard to *x_ei_* is expressed as:

(10) $$\frac{\partial K}{\partial x_{\mathrm{ei}}}u+K\frac{\partial u}{\partial x_{\mathrm{ei}}}=0$$

The partial derivative of the objective output displacement *u*_out_ can be deduced by introducing an indeterminate vector $\Lambda_{m}$ and an ancillary vector $\alpha_{m}$. In addition, the introduced two vectors can satisfy the subsequent adjoint relationship:

(11) $$K\Lambda_{m}=\alpha_{m}$$

where $\alpha_{m}={\left[\underset{m-1}{\underbrace{0,\cdots ,0}},\underset{m}{\underbrace{1}},0,\cdots ,0\right]}^{T}$, the *m*-th component is set as 1, and the remaining components are 0. In that case, the *m* corresponds to the degree freedom of the key node where the objective output displacement is located. The partial derivative of the objective output displacement in regard to *x_ei_* is achieved:

(12) $$\frac{\partial u_{\mathrm{out}}}{\partial x_{ei}}=\alpha_{m}^{T}\frac{\partial u}{\partial x_{ei}}=\alpha_{m}^{T}\frac{\partial u}{\partial x_{ei}}-\Lambda_{m}^{T}(\frac{\partial K}{\partial x_{ei}}u+K\frac{\partial u}{\partial x_{ei}})=(\alpha_{m}^{T}-\Lambda_{m}^{T}K)\frac{\partial u}{\partial x_{ei}}-\Lambda_{m}^{T}\frac{\partial K}{\partial x_{ei}}u=-\Lambda_{m}^{T}\frac{\partial K}{\partial x_{ei}}u$$

According to Equation (6), the global stiffness matrix ***K*** can be acquired:

(13) $$K=\sum_{i=1}^{N}k_{i}(x_{1i},x_{1i},\cdots x_{Ji})=\sum_{i=1}^{N}\left\{\sum_{j=2}^{J}\left[(k_{j}^{0}-k_{j-1}^{0})\prod_{q=1}^{j}f_{k}(x_{qi})\right]+f(x_{1i})k_{1}^{0}\right\}$$

As a result, the partial derivative of global stiffness matrix in regard to *x_ei_* is obtained:

(14) $$\frac{\partial K}{\partial x_{ei}}=\left\{\begin{array}{l} \sum_{j=2}^{J}\left\{(k_{j}^{0}-k_{j-1}^{0})\left[px_{1i}^{p-1}\prod_{q=2}^{j}f_{k}(x_{qi})\right]\right\}+px_{1i}^{p-1}k_{1}^{0},\;\;e=1\\ \sum_{j=e}^{J}\left\{(k_{j}^{0}-k_{j-1}^{0})\left[px_{ei}^{p-1}\prod_{q=1,q\neq e}^{j}f_{k}(x_{qi})\right]\right\},\;\;e=2,3,\ldots ,J \end{array}\right.$$

Substituting Equation (14) into Equation (12) generates:

(15) $$\frac{\partial u_{\mathrm{out}}}{\partial x_{ei}}=\left\{\begin{array}{l} -\Lambda_{m}^{T}\left\{\sum_{j=2}^{J}\left\{(k_{j}^{0}-k_{j-1}^{0})\left[px_{1i}^{p-1}\prod_{q=2}^{j}f_{k}(x_{qi})\right]\right\}+px_{1i}^{p-1}k_{1}^{0}\right\}u,\;\;e=1\\ -\Lambda_{m}^{T}\left\{\sum_{j=e}^{J}\left\{(k_{j}^{0}-k_{j-1}^{0})\left[px_{ei}^{p-1}\prod_{q=1,q\neq e}^{j}f_{k}(x_{qi})\right]\right\}\right\}u,\;\;e=2,3,\ldots ,J \end{array}\right.$$

The first-order partial derivative of the real volume in regard to the whole topological variables can be conveniently obtained using Equations (5), (7), and (8).

(16) $$\frac{\partial V}{\partial x_{ei}}=\left\{\begin{array}{l} \sum_{j=2}^{J}\left\{(v_{j}^{0}-v_{j-1}^{0})\left[\prod_{q=2}^{j}f_{v}(x_{qi})\right]\right\}+v_{1}^{0},\;\;e=1\\ \sum_{j=e}^{J}\left\{(v_{j}^{0}-v_{j-1}^{0})\left[\prod_{q=1,q\neq e}^{m}f_{v}(x_{qi})\right]\right\},\;\;e=2,3,\ldots ,J \end{array}\right.$$

The objective output displacement is approximated via first-order Taylor expansion:

(17) $$u_{\mathrm{out}}\approx u_{\mathrm{out}}^{\left(b\right)}+\sum_{e=1}^{J}\sum_{i=1}^{N}\frac{\partial u_{\mathrm{out}}}{\partial x_{ei}}\left|{}_{b}\left(x_{ei}-x_{ei}^{\left(b\right)}\right)\right.$$

where the symbol ‘*b*’ means the *b*-th optimization iteration.

Analogously, the real volume of the structure can also be formulated as follows:

(18) $$V\approx V_{}^{\left(b\right)}+\sum_{e=1}^{J}\sum_{i=1}^{N}\frac{\partial V}{\partial x_{ei}}\left|{}_{b}\left(x_{ei}-x_{ei}^{\left(b\right)}\right)\right.$$

Through the above derivation process, the complex Equation (7) can be converted into a classical linear programming (LP) problem. Finally, the convergent criterion is set as follows:

(19) $$\frac{\left|u_{\mathrm{out}}^{\left(b+1\right)}-u_{\mathrm{out}}^{\left(b\right)}\right|}{u_{\mathrm{out}}^{\left(b\right)}}\leq \xi$$

where ξ represents the convergent precision. The optimization iteration is terminated when ξ=0.001.

**Figure 3:**
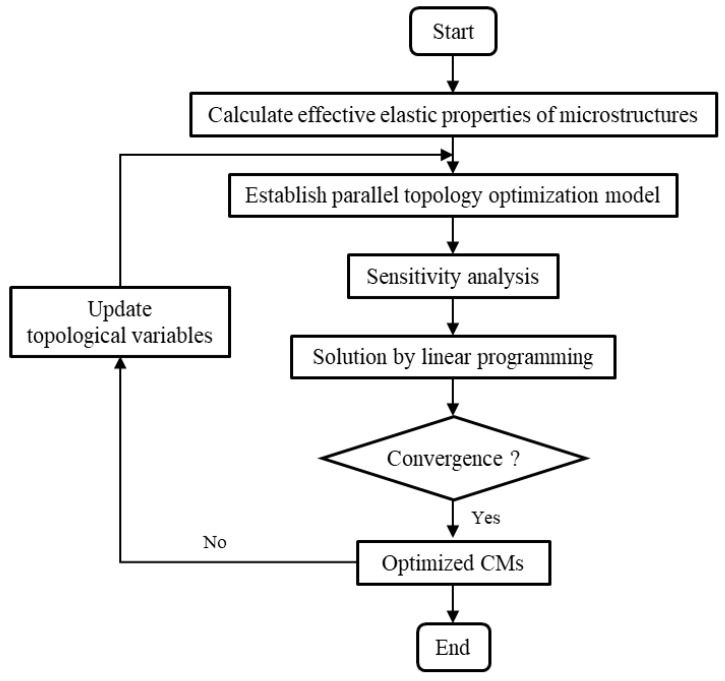
Flow chart of optimization process.

The topology optimization model was established and solved. Figure 3 displays a flow chart of optimization process to understand the proposed method conveniently.

## 4. Numerical Examples

The displacement inverter mechanism (DIM) and compliant gripper mechanism (CGM) are demonstrated to verify the feasibility of the present method in this section. Dimensionless units are utilized to facilitate the relevant discussion for material properties, geometric sizes, and external loads. Because optimization results depend on how to assign microstructures to the topological variables, an interpolation strategy is listed in Table 3 to select the microstructures.

### 4.1. Displacement Inverter Mechanism

**Figure 4:**
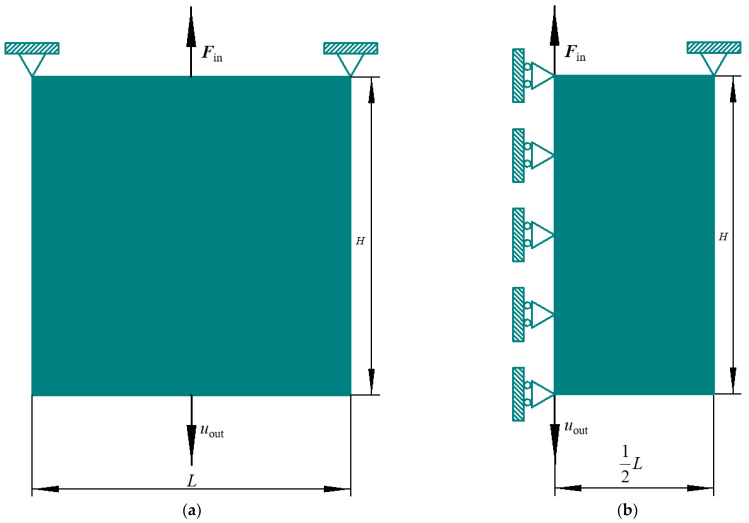
Original design domain for DIM: (**a**) whole design domain; (**b**) right half part.

The DIM is selected as the first example, and its design domain is exhibited in Figure 4. The geometric sizes of the original design domain are ***L*** = 240 and ***H*** = 240, with a thickness of 1. The upper left and right corners are fixed. At the midpoint of the top margin, a unit load ***F***_in_ is imposed straight upward. A mesh with 60 × 60 elements is adopted by dividing the original design domain. Due to the DIM being symmetrical, we only consider the right half domain. The aim is to maximize the downward displacement at the midpoint of the bottom margin under a prescribed total volume fraction constraint ***R***_v_ = 0.3.

**Figure 5:**
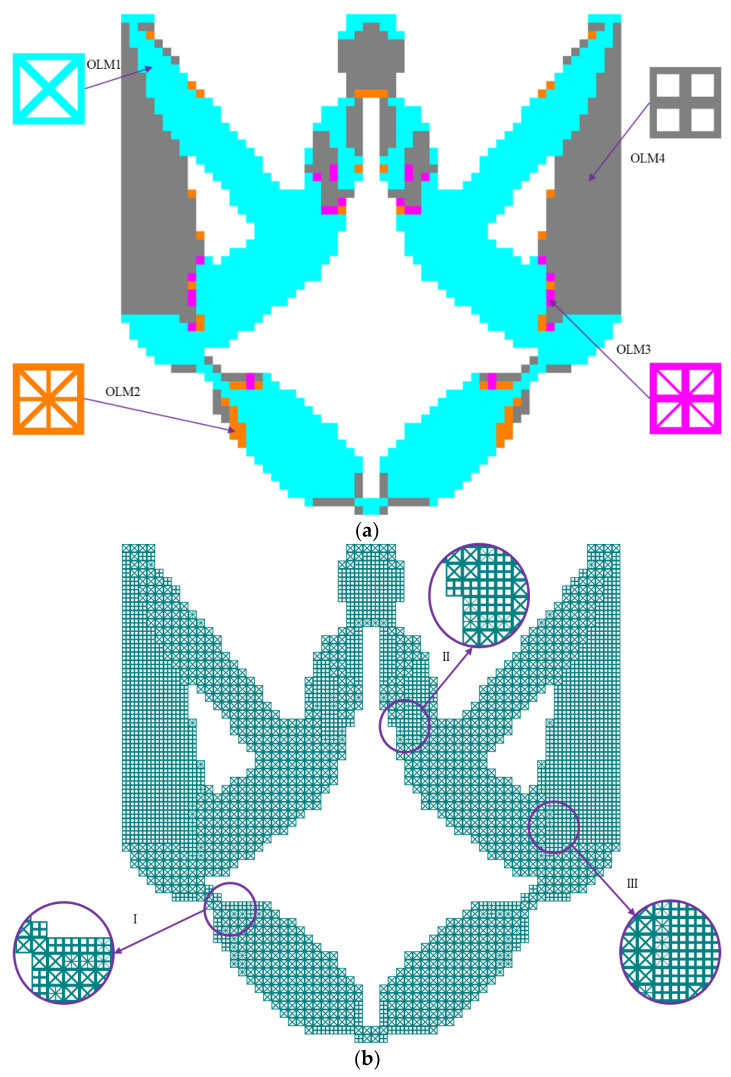
Optimized result of the DIM: (**a**) different OLMs’ distribution; (**b**) optimized HL-DIM with partial enlargement areas I, II and III.

**Figure 6:**
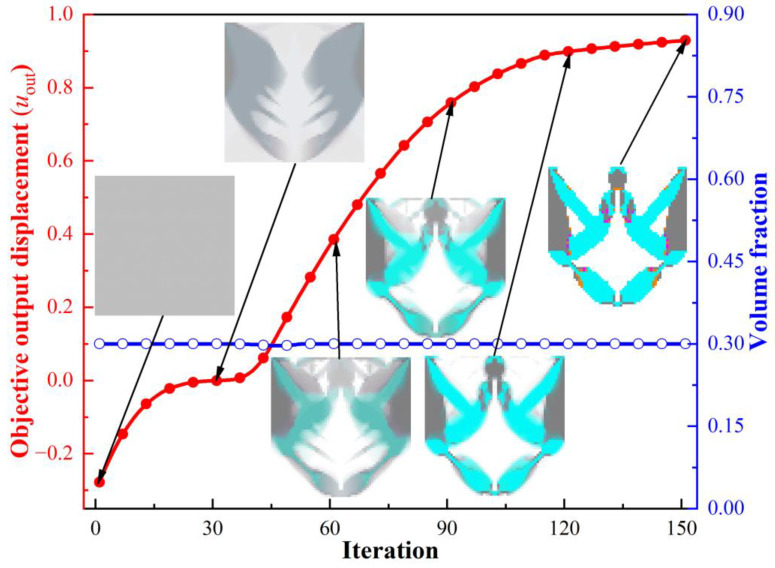
Iteration histories of objective output displacement and volume faction of the HL-DIM.

The distribution of multiple OLMs and the optimized hybrid lattice DIM (HL-DIM) are displayed in Figure 5. Lattice microstructures with various configurations are distributed in designated locations to play more significant roles across the overall design domain. As depicted in Figure 5a, OLM1 is primarily utilized in the cyanine regions, which require the relatively most compliant effective elastic properties; OLM4 with the relatively stiffest vertical or horizontal effective elastic properties is located in the gray regions. Furthermore, small quantities of OLM2 with inferior most compliant effective elastic properties and OLM3 with subordinate stiffest vertical or horizontal effective elastic properties are separately placed in the transitional orange and magenta regions. The partial enlargement areas I-III in Figure 5b illustrate that the gradual change of different lattice microstructures in the translational regions can be convenient to fulfill better compliant performance. Various lattice microstructures increase the design freedom. Combining the sensitivity filter, the corner-to-corner joints are avoided. Figure 6 demonstrates the iteration histories of the objective output displacements and volume fraction for the HL-DIM with OLMs, in which the intermediate topological evolution facilitates the understanding of the progress of the optimization.

**Figure 7:**
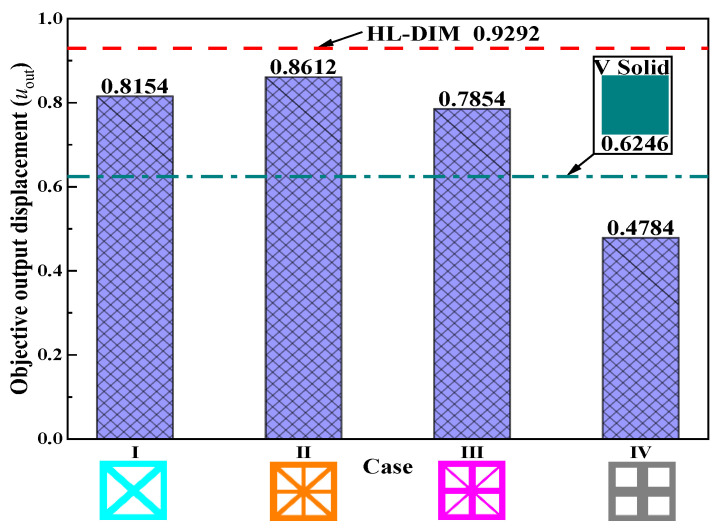
Objective output displacement comparison for different DIMs.

In order to reveal the superiorities of the HL-DIM, four kinds of uniform lattice DIMs (UL-DIMs) and a traditional solid DIM are presented for comparison. These DIMs are composed of uniform lattice microstructures or solid microstructures. Various types of DIMs and their corresponding objective displacements are listed in Table 4. The microstructure configurations have an evident influence on the topological shapes of DIMs and their corresponding output displacements. Obviously, Case II has the largest output displacement, 80.02% larger than Case IV, which obtains the smallest output displacement. These samples apparently demonstrate that the OLM2 in Case II commendably balances the compliant and stiff effective elastic properties. However, the objective output displacements acquired by Cases I–V are suboptimal compared with the HL-DIM, as exhibited in Figure 7. The optimized HL-DIM in Figure 5 possesses a better compliant effect with an objective output displacement ***u***_out_ = 0.9292. Its objective output displacement exceeds the objective displacements of Cases I–V by 13.96%, 7.90%, 18.31%, 94.23%, and 48.77%, respectively. Attractively, the compliant effect of Case V (solid DIM) is inferior to that of Cases I–III, but superior to that of Case IV. This situation indicates that the OLM4 in Case IV is not suitable for the UL-DIMs design. The reason for this may be that the OLM4 only guarantees the relatively stiffest horizontal or vertical effective elastic properties, but it could not coordinate the compliant and stiff effective elastic properties. On account that the design freedom increases, the HL-DIM consisting of various OLMs has a superior compliant effect compared to the UL-DIM comprising a single OLM and the traditional DIM with a solid microstructure. This example shows that the ICM method is suitable for the design of HLCMs and can greatly improve the compliant performance.

**Figure 8:**
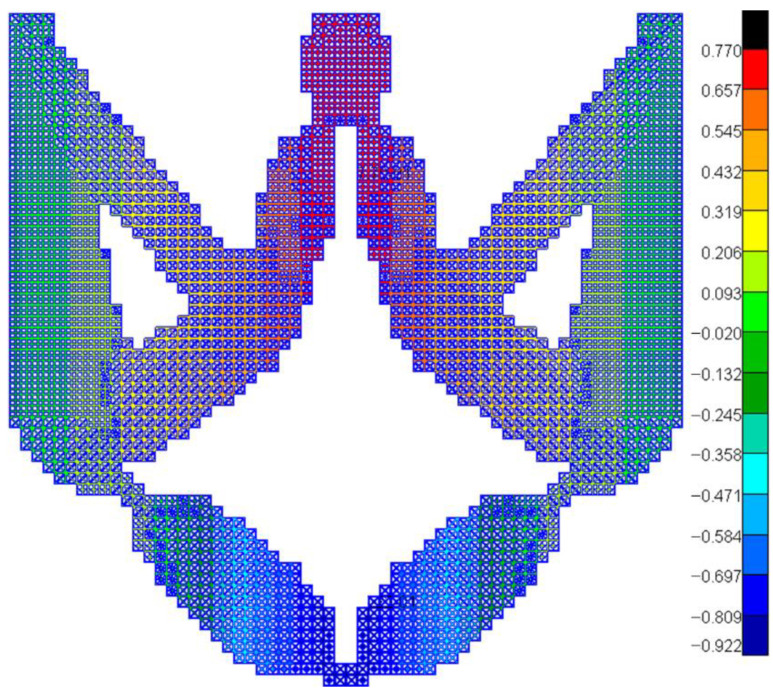
Vertical deformation fringe for HL-DIM with OLMs.

Besides, in order to confirm the effectiveness of the optimized result with homogenization theory, the HL-DIM with OLMs in Figure 5b is chosen to complete the finite element simulation without homogenization theory. Figure 8 exhibits the vertical deformation fringe. The objective output displacement in numerical simulation is 0.9048, and the objective output displacement with homogenization theory is 0.9292. The relative error is merely 2.70%, which validates that the present method is effective.

### 4.2. Compliant Gripper Mechanism

**Figure 9:**
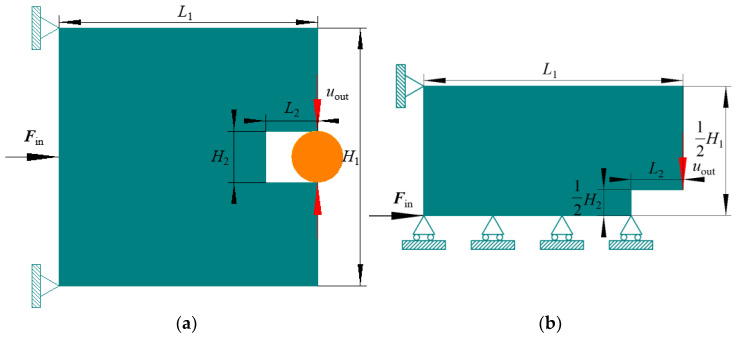
Original design domain for CGM: (**a**) whole design domain; (**b**) upper half part.

A CGM in Figure 9 is exhibited as the second benchmark. The geometrical sizes contain ***L***_1_ = 200, ***H***_1_ = 200, ***L***_2_ = 40, and ***H***_2_ = 40, and there is a thickness of 1. The upper and lower left corners are fastened. At the midpoint of the left margin, a unit load *****F*****_in_ is imposed horizontally rightward. Due to the CGM also being symmetrical, we only consider the upper half part. The aim is to make the downward displacement maximization at the lower right corner of the upper half domain under a prescribed total volume fraction constraint ***R***_v_ = 0.3.

**Figure 10:**
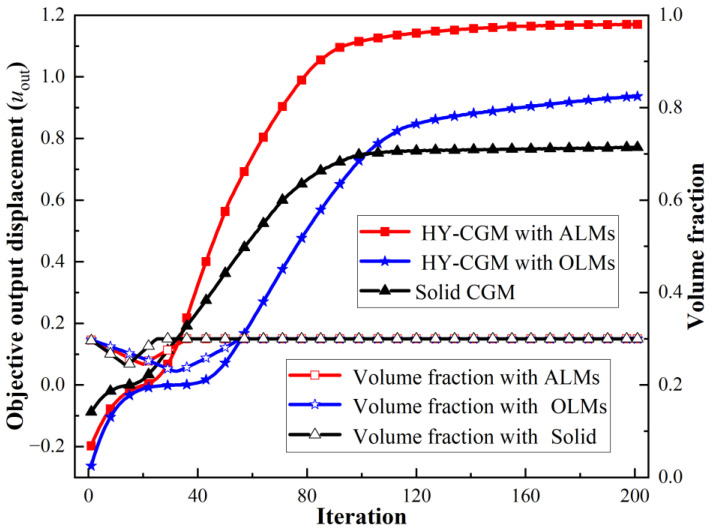
Iteration histories of objective output displacement and volume faction of the CGMs.

**Figure 11:**
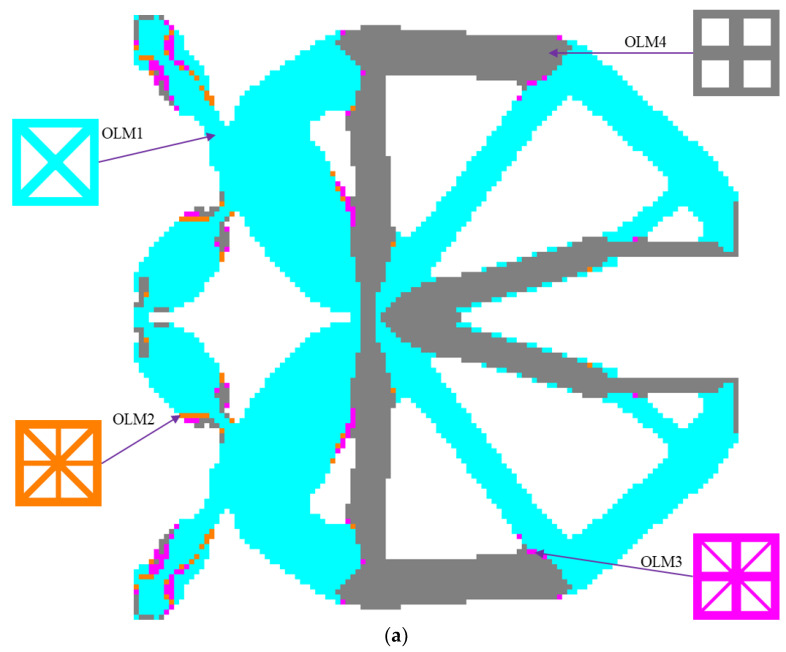

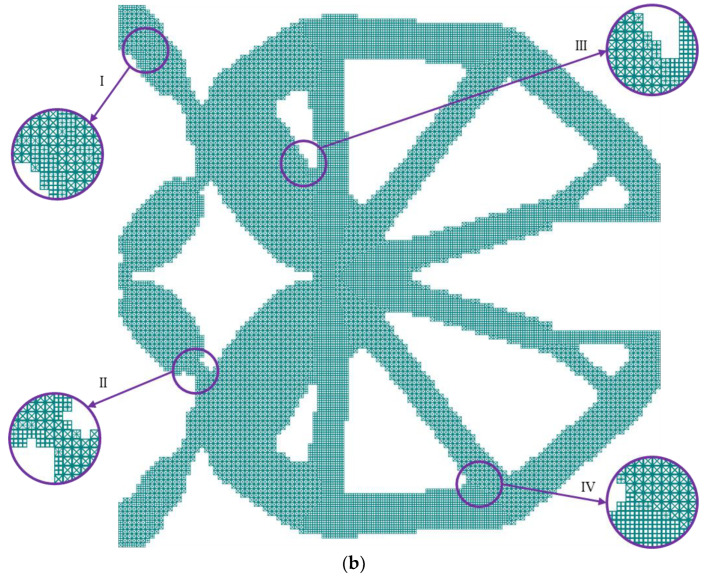
Optimized result with OLMs for the CGM: (**a**) layout of different OLMs; (**b**) optimized orthogonal HL-CGM with partial enlargement areas I, II, III and IV.

**Figure 12:**
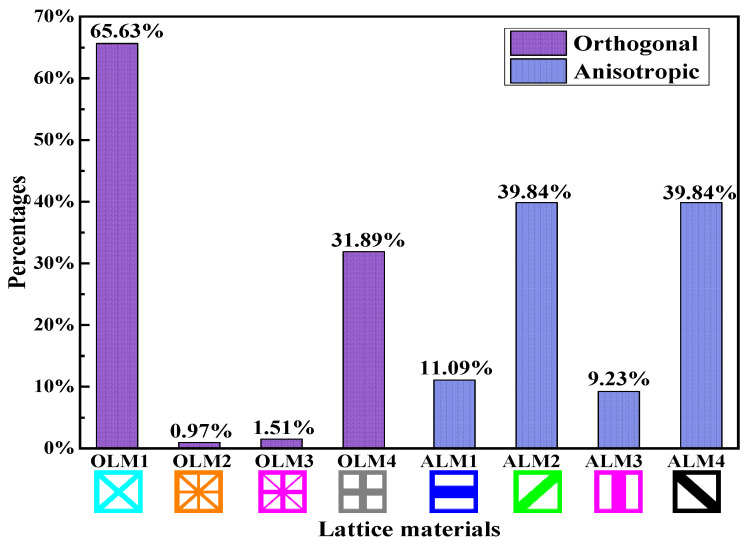
OLMs and ALMs percentages of HY-CGMs.

Figure 10 demonstrates the iteration histories of the objective output displacements and volume fractions for the solid CGM and hybrid lattice CGMs (HL-CGMs) with OLMs or ALMs. The objective output displacements increase quickly in the initial stage; then go through a short, slowly increasing plateau stage on account of negative displacements transforming to positive displacements; and finally continue to increase until they converge to 0.7714 (solid CGM), 0.9366 (HL-CGM with OLMs), and 1.1702 (HL-CGM with ALMs). Noticeably, the volume fractions remain fairly stable except for the relating initial stage and short plateau stage, which always meet the constraints during the iterative process. The optimized HL-CGM with different OLMs is shown in Figure 11. A similar situation to Figure 5 is observed. The cyanine regions distribute OLM1, which possesses the relatively most compliant effective elastic properties. The gray regions are occupied by OLM4 with the relatively stiffest horizontal or vertical effective elastic properties. Besides, the transitional orange regions and magenta regions separately feature small quantities of OLM2 with inferior most compliant effective elastic properties and OLM3 with subordinate stiffest vertical or horizontal effective elastic properties. The partial enlargement areas I–IV in Figure 11b show that the gradual change of different OLMs in the translational regions could be helpful to realize better compliant performance. The percentages of various OLMs in Figure 11b are shown in Figure 12. OLM1 and OLM4 account for 65.63% and 31.89%, respectively, but the proportions of OLM2 and OLM3 with eclectic effective mechanical properties are merely 0.97% and 1.51%, respectively. This indicates that only OLM1 and OLM4 play a leading role, whereas the compromised OLM2 and OLM3 could not be fully utilized.

**Figure 13:**
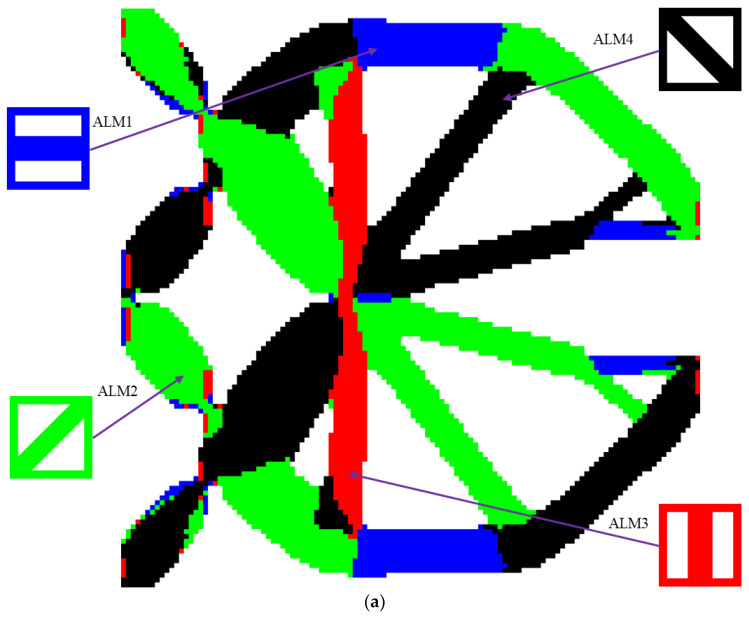

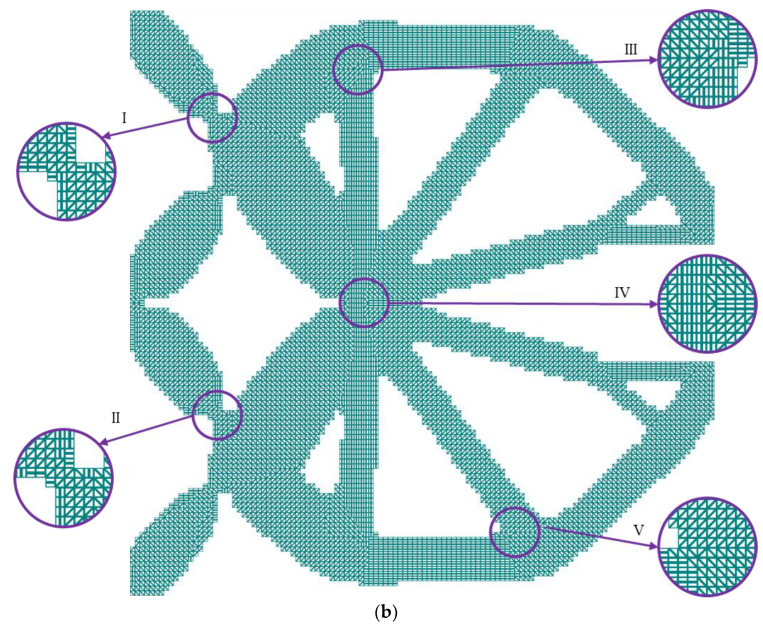
Optimized result with ALMs for the CGM: (**a**) layout of different ALMs; (**b**) optimized anisotropic HL-CGM with partial enlargement areas I, II, III, IV and V.

In order for all different types of microstructures to play important roles and take full advantage of the constitutive materials, ALM1-ALM4 are applied to design the CGM. Figure 13 exhibits the layout of the different ALMs and the optimized anisotropic HL-CGM. ALM1 is primarily utilized in the blue regions, which demand the relatively most superior horizontal mechanical properties. ALM3 is mainly placed in the red regions, which demand the relatively most superior vertical mechanical properties. ALM2 and ALM4 with relatively excellent shear resistance properties separately distribute in the green and black regions. The partial enlargement areas I–V in Figure 13b show that the delicate layout of different ALMs could be convenient to fulfill better compliant performance than the OLMs by means of gradient changes in the translational regions. The percentages of various ALMs in Figure 13b are also shown in Figure 12. ALM1 and ALM3 account for 11.09% and 9.23%, respectively. In view of the symmetry of the CGM, the percentages of ALM2 and ALM4 are identical at 39.84%. For the HL-CGM with ALMs in Figure 13, each ALM accounts for more than 9%. This situation indicates that ALM1, ALM2, ALM3, and ALM4 make significant contributions to the compliant performance and are efficiently utilized. In contrast to the HL-CGM with OLMs in Figure 11b, the HL-CGM with ALMs in Figure 13b is superior. The objective output displacement of HL-CGM with ALMs is 1.1702, exceeding the former by 24.94%. The compliant performance of the HL-CGM with ALMs is obviously superior to that of the HL-CGM with OLMs.

**Figure 14:**
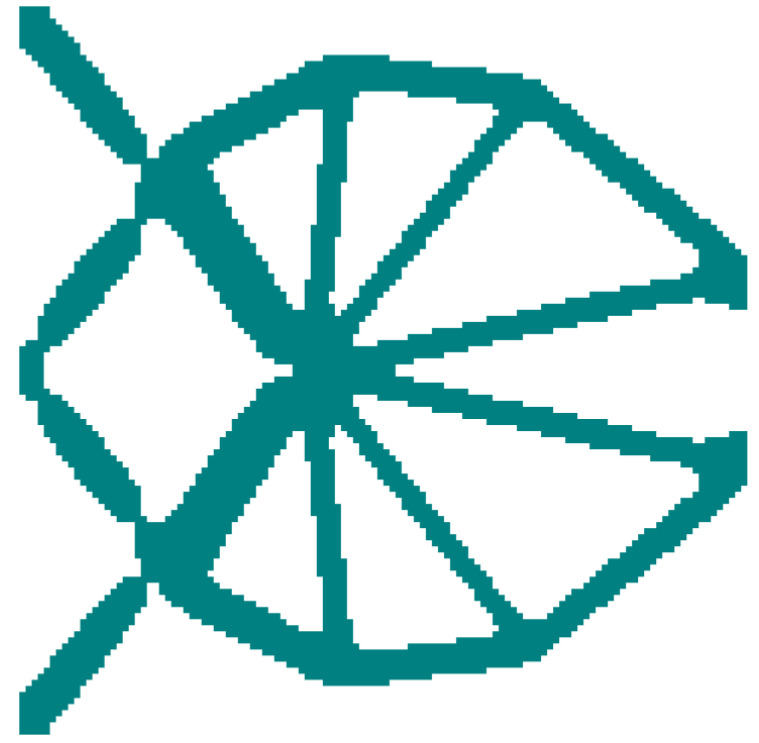
Optimized solid CGM.

**Figure 15:**
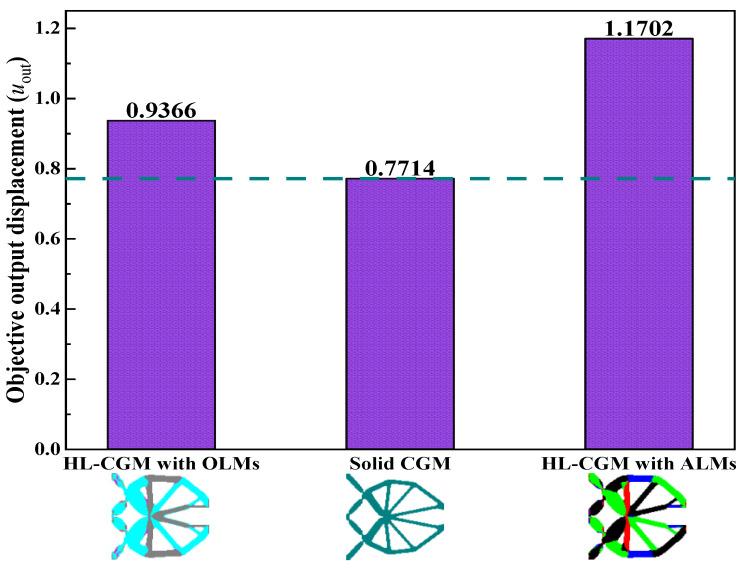
Objective output displacement comparison of different CGMs.

Moreover, a traditional optimized CGM with only solid material is designed for comparison with the abovementioned HL-CGMs with OLMs and ALMs by constraining the equal volume fractions. The optimized solid CGM is displayed in Figure 14. The objective output displacements acquired by the solid CGM are inferior compared with the HL-CGMs with OLMs and ALMs, as exhibited in Figure 15. The optimized HL-CGMs with OLMs or ALMs in Figure 11 and Figure 13 possess superior compliant effects with objective output displacements of 0.9366 and 1.1702, respectively. Their objective output displacements exceed those of the solid CGM by 21.42% and 51.70%, respectively. This example shows that the ALMs could make full use of constitutive materials, thereby significantly improving the compliant performance of the CGM based on the ICM method.

To reveal the superiorities of the HL-CGM with ALMs, two kinds of uniform lattice CGMs (UL-CGMs) with ALM1 or ALM3 are presented for comparison. Because the design domain of CGM is transversely symmetrical, only the upper half part is considered in the optimization process. Because of ALM2 and ALM4 being transversely asymmetrical, UL-CGMs with ALM2 or ALM4 are unreasonable. Therefore, two kinds of HL-CGMs with ALM2 and ALM4 are also proposed for comparison. Various types of CGMs and their corresponding objective displacements are listed in Table 5. The objective displacements of Cases I–IV are 0.7509, 0.8866, 0.6133, and 0.8580, respectively. The microstructure configurations have an evident influence on the topological shapes of CGMs and their corresponding output displacements. Apparently, Case II has the largest output displacement, 44.56% larger than Case III, which obtains the smallest output displacement. However, the objective output displacements acquired by Cases I–IV are suboptimal compared with the HL-CGM in Figure 13, the objective output displacement of which is 1.1702 and exceeds the objective displacements of Cases I–IV by 55.84%, 37.99%, 90.80%, and 36.39%, respectively. This situation indicates that the ALM3 in Case III is not suitable for the UL-CGMs design. The reason for this may be that the ALM3 only guarantees the relatively stiffest vertical effective elastic properties, but it cannot adapt to the macroscale topology. The HL-CGM consisting of ALM1, ALM2, ALM3, and ALM4 has a superior compliant effect compared to the UL-CGM comprising a single ALM1 or ALM3 and the HL-CGM with only ALM2 and ALM4.

## 5. Conclusions

This paper proposes a novel optimization method to design HLCMs with different microstructures via a generalized multi-material-recognizing interpolation scheme using the ICM method. The effective properties of multiple OLMs and ALMs were calculated by means of the homogenization theory to coordinate the layout of the macroscale structure and the selection of various lattice microstructures. The pivotal filter functions facilitated achieving the multi-material modeling due to the independent continuous variables being convenient to characterize different kinds of element properties. Sensitivity analysis and linear programming were utilized to solve the parallel topology optimization model. Some numerical examples were introduced to demonstrate the validation of the present method. Compared with UL-DIMs with a single microstructure, HL-DIMs presented a superior compliant performance. In addition, HL-CGMs with various ALMs possessed a superior compliant performance to those with different OLMs, revealing that ALMs could make full use of the constitutive material and contribute to the improvement of the compliant performance. This method offers a significant reference to optimize HLCMs and promotes the development of the ICM method.

## Figures and Tables

**Table 1:** Effective elastic tensors of OLMs.

OLMs	*r*	*s*	Configurations	Effective Elastic Tensor *D*^H^
OLM1	0	0.12 *w*	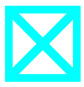	$\left[\begin{array}{ccc} 325.10 & 106.15 & 0\\ 106.15 & 325.10 & 0\\ 0 & 0 & 103.69 \end{array}\right]$
OLM2	0.06 *w*	0.08 *w*	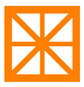	$\left[\begin{array}{ccc} 350.97 & 86.81 & 0\\ 86.81 & 350.97 & 0\\ 0 & 0 & 77.22 \end{array}\right]$
OLM3	0.11 *w*	0.04 *w*	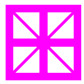	$\left[\begin{array}{ccc} 369.17 & 72.70 & 0\\ 72.70 & 369.17 & 0\\ 0 & 0 & 61.58 \end{array}\right]$
OLM4	0.17 *w*	0	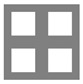	$\left[\begin{array}{ccc} 399.15 & 56.41 & 0\\ 56.41 & 399.15 & 0\\ 0 & 0 & 37.05 \end{array}\right]$

**Table 2:** Effective elastic tensors of ALMs.

ALMs	*t*	*θ* (^o^)	Configurations	Effective Elastic Tensor *D*^H^
ALM1	0.30 *w*	0	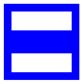	$\left[\begin{array}{ccc} 519.49 & 41.14 & 0\\ 41.14 & 223.79 & 0\\ 0 & 0 & 22.62 \end{array}\right]$
ALM2	0.24 *w*	45	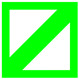	$\left[\begin{array}{ccc} 298.15 & 85.47 & 72.87\\ 85.47 & 298.15 & 72.87\\ 72.87 & 72.87 & 105.33 \end{array}\right]$
ALM3	0.30 *w*	90	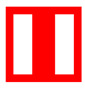	$\left[\begin{array}{ccc} 223.79 & 41.14 & 0\\ 41.14 & 519.49 & 0\\ 0 & 0 & 22.62 \end{array}\right]$
ALM4	0.24 *w*	135	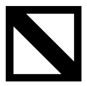	$\left[\begin{array}{ccc} 298.15 & 85.47 & -72.87\\ 85.47 & 298.15 & -72.87\\ -72.87 & -72.87 & 105.33 \end{array}\right]$

**Table 3:** Adopted interpolation strategy.

*x*_1*i*_	*x*_2*i*_	*x*_3*i*_	*x*_4*i*_	Selection
0.0001	-	-	-	void
1	0	-	-	OLM1
1	1	0	-	OLM2
1	1	1	0	OLM3
1	1	1	1	OLM4

**Table 4:** Uniform DIMs with different lattice microstructures.

Case	Material	Microstructures	DIM	Objective Displacement *u*_out_
I	OLM1	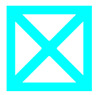	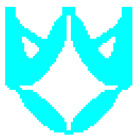	0.8154
II	OLM2	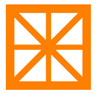	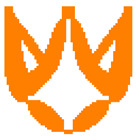	0.8612
III	OLM3	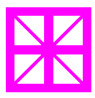	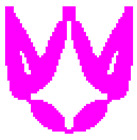	0.7854
IV	OLM4	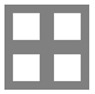	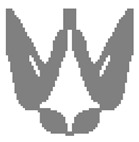	0.4784
V	Solid	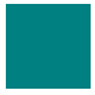	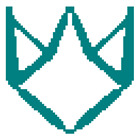	0.6246

**Table 5:** CGMs with different lattice microstructures.

Case	Material	Microstructures	CGM	Objective Displacement *u*_out_
I	ALM1	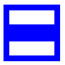	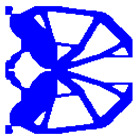	0.7509
II	ALM2 + ALM4	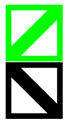	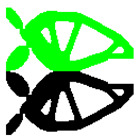	0.8866
III	ALM3	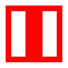	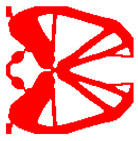	0.6133
IV	ALM4 + ALM2	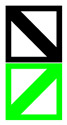	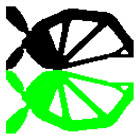	0.8580

## Data Availability

Not applicable.

## References

[1] Zhu B., Zhang X., Zhang H., Liang J., Zang H., Li H., Wang R. (2020). Design of Compliant Mechanisms Using Continuum Topology Optimization: A Review. Mech. Mach. Theory.

[2] Verotti M., Berselli G., Bruzzone L., Baggetta M., Fanghella P. (2021). Design, Simulation and Testing of an Isotropic Compliant Mechanism. Precis. Eng..

[3] Liu Y., Zhang Y., Xu Q. (2017). Design and Control of a Novel Compliant Constant-Force Gripper Based on Buckled Fixed-Guided Beams. IEEE/ASME Trans. Mechatron..

[4] Tian Y., Zhang D., Shirinzadeh B. (2011). Dynamic Modelling of a Flexure-Based Mechanism for Ultra-Precision Grinding Operation. Precis. Eng..

[5] Ando B., Baglio S., L’Episcopo G., Trigona C. (2012). Investigation on Mechanically Bistable Mems Devices for Energy Harvesting From Vibrations. J. Microelectromech. Syst..

[6] Cirelli M., Cera M., Pennestrì E., Valentini P.P. (2020). Nonlinear Design Analysis of Centrifugal Pendulum Vibration Absorbers: An Intrinsic Geometry-Based Framework. Nonlinear Dyn..

[7] Zhao H., Zhao C., Ren S., Bi S. (2019). Analysis and Evaluation of a Near-Zero Stiffness Rotational Flexural Pivot. Mech. Mach. Theory.

[8] Dearden J., Grames C., Orr J., Jensen B.D., Magleby S.P., Howell L.L. (2018). Cylindrical Cross-Axis Flexural Pivots. Precis. Eng..

[9] Krishnan G., Kim C., Kota S. (2011). An Intrinsic Geometric Framework for the Building Block Synthesis of Single Point Compliant Mechanisms. J. Mech. Robot..

[10] Kim C.J., Moon Y., Kota S. (2008). A Building Block Approach to the Conceptual Synthesis of Compliant Mechanisms Utilizing Compliance and Stiffness Ellipsoids. J. Mech. Des..

[11] Sigmund O. (1997). On the Design of Compliant Mechanisms Using Topology Optimization. Mech. Struct. Mach. Int. J..

[12] Tran A.V., Zhang X., Zhu B. (2018). The Development of a New Piezoresistive Pressure Sensor for Low Pressures. IEEE Trans. Ind. Electron..

[13] Cao L., Dolovich A.T., Chen A., Zhang W.C. (2018). Topology Optimization of Efficient and Strong Hybrid Compliant Mechanisms Using a Mixed Mesh of Beams and Flexure Hinges with Strength Control. Mech. Mach. Theory.

[14] Wu J., Sigmund O., Groen J.P. (2021). Topology Optimization of Multi-Scale Structures: A Review. Struct. Multidiscip. Optim..

[15] Bendsøe M.P. (1989). Optimal Shape Design as a Material Distribution Problem. Struct. Optim..

[16] Groen J.P., Langelaar M., Sigmund O., Ruess M. (2017). Higher-Order Multi-Resolution Topology Optimization Using the Finite Cell Method. Int. J. Numer. Meth. Eng..

[17] Bendsøe M.P., Kikuchi N. (1988). Generating Optimal Topologies in Structural Design Using a Homogenization Method. Comput. Method. Appl. M..

[18] Ye M., Gao L., Wang F., Li H. (2021). A Novel Design Method for Energy Absorption Property of Chiral Mechanical Metamaterials. Materials.

[19] Zheng J., Luo Z., Jiang C., Gao J. (2019). Robust Topology Optimization for Concurrent Design of Dynamic Structures Under Hybrid Uncertainties. Mech. Syst. Signal Process..

[20] Wang M.Y., Wang X., Guo D. (2003). A Level Set Method for Structural Topology Optimization. Comput. Methods Appl. Mech. Eng..

[21] Sun Z., Song Z., Song J., Li H., Guo X. (2022). Structural Optimization of Fiber-Reinforced Material Based On Moving Morphable Components (Mmcs). Acta Mech. Solida Sin..

[22] Guo X., Zhang W., Zhong W. (2014). Doing Topology Optimization Explicitly and Geometrically—A New Moving Morphable Components Based Framework. J. Appl. Mech..

[23] Xie Y.M., Steven G.P. (1993). A Simple Evolutionary Procedure for Structural Optimization. Comput. Struct..

[24] Xue L., Wen G., Wang H., Liu J. (2022). Eigenvectors-Guided Topology Optimization to Control the Mode Shape and Suppress the Vibration of the Multi-Material Plate. Comput. Method Appl. Mech. Eng..

[25] Carraturo M., Rocca E., Bonetti E., Hömberg D., Reali A., Auricchio F. (2019). Graded-Material Design Based On Phase-Field and Topology Optimization. Comput. Mech..

[26] Takezawa A., Nishiwaki S., Kitamura M. (2010). Shape and Topology Optimization Based On the Phase Field Method and Sensitivity Analysis. J. Comput. Phys..

[27] Zhang X., Ye H., Wei N., Tao R., Luo Z. (2021). Design Optimization of Multifunctional Metamaterials with Tunable Thermal Expansion and Phononic Bandgap. Mater. Des..

[28] Wei N., Ye H., Zhang X., Wang W., Sui Y. (2022). Lightweight Topology Optimization of Graded Lattice Structures with Displacement Constraints Based On an Independent Continuous Mapping Method. Acta Mech. Sin..

[29] Nishiwaki S., Frecker M.I., Min S., Kikuchi N. (1998). Topology Optimization of Compliant Mechanisms Using the Homogenization Method. Int. J. Numer. Meth. Eng..

[30] Larsen U.D., Sigmund O., Bouwstra S. (1997). Design and Fabrication of Compliant Micromechanisms and Structures with Negative Poisson’s Ratio. J. Microelectromech. Syst..

[31] Sigmund O. (2001). Design of Multiphysics Actuators Using Topology Optimization—Part II: Two-Material Structures. Comput. Methods Appl. Mech. Eng..

[32] Wang N.F., Hu K., Zhang X.M. (2017). Hierarchical Optimization for Topology Design of Multi-Material Compliant Mechanisms. Eng. Optimiz..

[33] Gaynor G.T., Meisel N.A., Williams C.B., Guest J.K. (2014). Multiple-Material Topology Optimization of Compliant Mechanisms Created Via Polyjet Three-Dimensional Printing. J. Manuf. Sci. Eng..

[34] Zuo W., Saitou K. (2017). Multi-Material Topology Optimization Using Ordered Simp Interpolation. Struct. Multidiscip. Optim..

[35] Chu S., Gao L., Xiao M., Luo Z., Li H. (2018). Stress-Based Multi-Material Topology Optimization of Compliant Mechanisms. Int. J. Numer. Meth. Eng..

[36] Wang Y., Wang M.Y., Chen F. (2016). Structure-Material Integrated Design by Level Sets. Struct. Multidiscip. Optim..

[37] Liu L., Yan J., Cheng G. (2008). Optimum Structure with Homogeneous Optimum Truss-Like Material. Comput. Struct..

[38] Rodrigues H., Guedes J.M., Bendsoe M.P. (2002). Hierarchical Optimization of Material and Structure. Struct. Multidiscip. Optim..

[39] Xia L., Breitkopf P. (2015). Multiscale Structural Topology Optimization with an Approximate Constitutive Model for Local Material Microstructure. Comput. Methods Appl. Mech. Eng..

[40] Sivapuram R., Dunning P.D., Kim H.A. (2016). Simultaneous Material and Structural Optimization by Multiscale Topology Optimization. Struct. Multidiscip. Optim..

[41] Vu-Huu T., Phung-Van P., Nguyen-Xuan H., Abdel Wahab M. (2018). A Polytree-Based Adaptive Polygonal Finite Element Method for Topology Optimization of Fluid-Submerged Breakwater Interaction. Comput. Math. Appl..

[42] Hoang V., Pham T., Tangaramvong S., Bordas S.P.A., Nguyen-Xuan H. (2021). Robust Adaptive Topology Optimization of Porous Infills Under Loading Uncertainties. Struct. Multidiscip. Optim..

[43] Hoang V., Pham T., Ho D., Nguyen-Xuan H. (2022). Robust Multiscale Design of Incompressible Multi-Materials Under Loading Uncertainties. Eng. Comput. Ger..

[44] Ngoc N.M., Hoang V., Lee D. (2022). Concurrent Topology Optimization of Coated Structure for Non-Homogeneous Materials Under Buckling Criteria. Eng. Comput. Ger..

[45] Merriam E.G., Tolman K.A., Howell L.L. (2016). Integration of Advanced Stiffness-Reduction Techniques Demonstrated in a 3D-Printable Joint. Mech. Mach. Theory.

[46] Merriam E.G., Howell L.L. (2016). Lattice Flexures: Geometries for Stiffness Reduction of Blade Flexures. Precis. Eng..

[47] Arredondo-Soto M., Cuan-Urquizo E., Gómez-Espinosa A. (2021). A Review On Tailoring Stiffness in Compliant Systems, Via Removing Material: Cellular Materials and Topology Optimization. Appl. Sci..

[48] Lee J., Kim D., Nomura T., Dede E.M., Yoo J. (2018). Topology Optimization for Continuous and Discrete Orientation Design of Functionally Graded Fiber-Reinforced Composite Structures. Compos. Struct..

[49] Conlan-Smith C., Bhattacharyya A., James K.A. (2018). Optimal Design of Compliant Mechanisms Using Functionally Graded Materials. Struct. Multidiscip. Optim..

[50] Tong X., Ge W., Zhang Y. (2017). Optimal Fiber Orientation and Topology Design for Compliant Mechanisms with Fiber-Reinforced Composites. Proc. Inst. Mech. Eng. Part C J. Mech. Eng. Sci..

[51] Liu Z., Xia L., Xia Q., Shi T. (2020). Data-Driven Design Approach to Hierarchical Hybrid Structures with Multiple Lattice Configurations. Struct. Multidiscip. Optim..

[52] Da D., Xia L. (2021). Design of Heterogeneous Mesostructures for Nonseparated Scales and Analysis of Size Effects. Int. J. Numer. Meth. Eng..

[53] Panettieri E., Boissin E., Montemurro M., Catapano A., Jalocha D. (2021). On the Accuracy of a Homogenized Continuum Model of Lattice Structures in Modal Analyses. Mech. Adv. Mater. Struc..

[54] Zhang H., Wu W., Kang Z., Feng X. (2020). Topology Optimization Method for the Design of Bioinspired Self-Similar Hierarchical Microstructures. Comput. Methods Appl. Mech. Eng..

[55] Andreassen E., Andreasen C.S. (2014). How to Determine Composite Material Properties Using Numerical Homogenization. Comp. Mater. Sci..

[56] Ye H., Dai Z., Wang W., Sui Y. (2019). ICM Method for Topology Optimization of Multimaterial Continuum Structure with Displacement Constraint. Acta Mech. Sin..

[57] Wei N., Ye H., Zhang X., Li J., Sui Y. (2022). Topology Optimization for Design of Hybrid Lattice Structures with Multiple Microstructure Configurations. Acta Mech. Solida Sin..

[58] Deng H., Vulimiri P.S., To A.C. (2022). An Efficient 146-Line 3D Sensitivity Analysis Code of Stress-Based Topology Optimization Written in Matlab. Optim. Eng..

[59] Chen F., Zhu J., Du X., Zhang R., Zhang W. (2022). Shape Preserving Topology Optimization for Structural Radar Cross Section Control. Chin. J. Aeronaut..

